# Supplementary figures and images for: Genetic alteration of histone lysine methyltransferases and their significance in renal cell carcinoma
Source: PeerJ. 2019 Feb 6;7:e6396. doi: 10.7717/peerj.6396 (PMC6368835; doi:10.7717/peerj.6396)

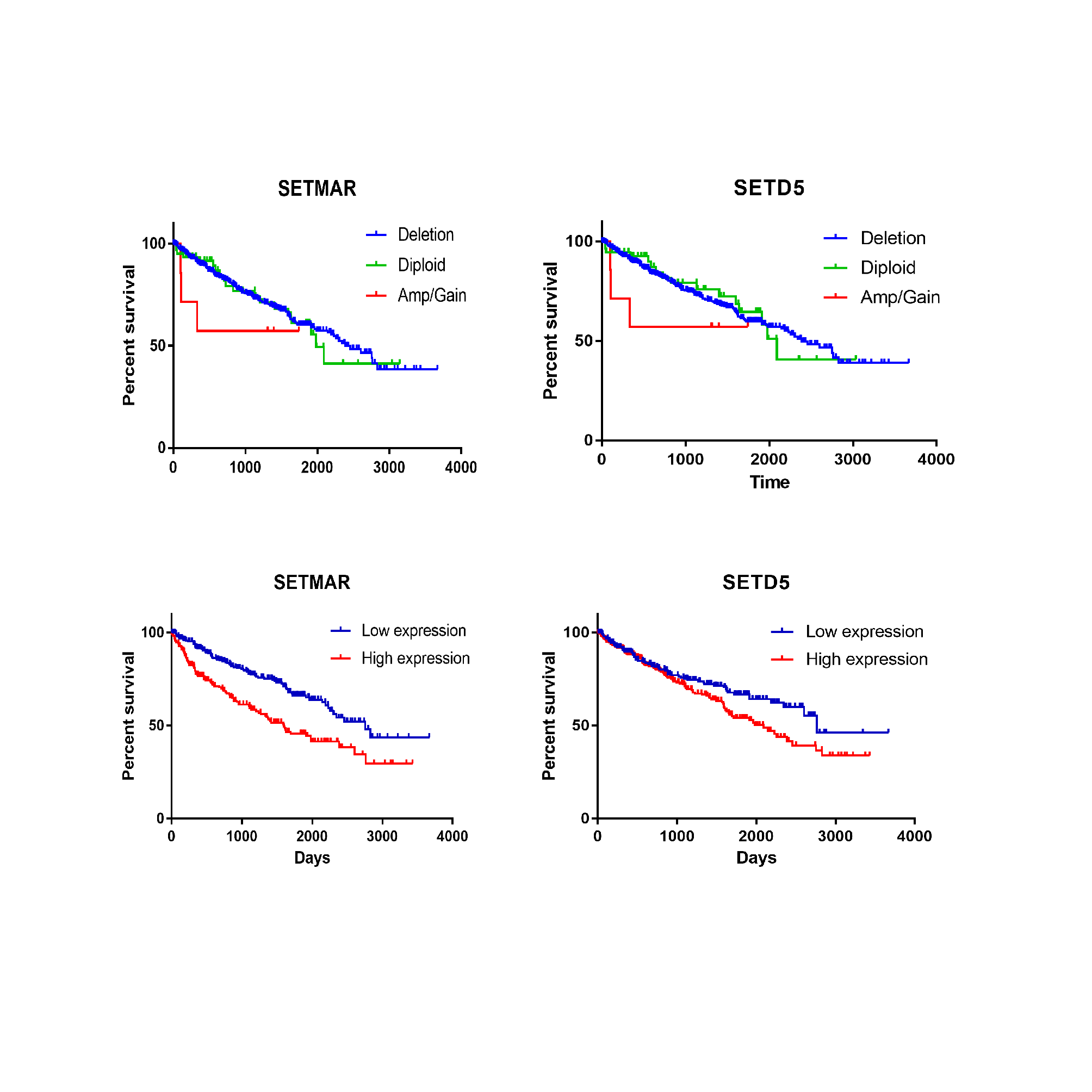

Supplement: Supplemental Information 1 — Kaplan-Meier plots of overall survival associated with copy number and mRNA expression levels of SETMAR and SETD5 in renal cell carcinoma. [file peerj-07-6396-s001.tif]
